# Comprehensive analysis of presurgical factors predicting psychiatric disorders in patients with refractory temporal lobe epilepsy and mesial temporal sclerosis underwent cortico‐amygdalohippocampectomy

**DOI:** 10.1002/jcla.22724

**Published:** 2018-12-05

**Authors:** Wei Yang, Chongyi Chen, Bo Wu, Qiaoyu Yang, Dongdong Tong

**Affiliations:** ^1^ Department of Neurosurgery Hospital of Chengdu Office of People’s Government of Tibetan Autonomous Region Chengdu China; ^2^ Department of Neurosurgery, West China Hospital Sichuan University Chengdu China

**Keywords:** cortico‐amygdalohippocampectomy, employed status, presurgical factors, psychiatric disorders, temporal lobe epilepsy‐mesial temporal sclerosis

## Abstract

**Background:**

This study aimed to evaluate the predictive value of presurgical factors for psychiatric disorders (PD) in refractory temporal lobe epilepsy and mesial temporal sclerosis (TLE‐MTS) patients underwent cortico‐amygdalohippocampectomy (CAH).

**Methods:**

A total of 98 refractory TLE‐MTS patients underwent CAH were consecutively enrolled in this cohort study. Several presurgical factors were recorded, such as married status, employment status, highest education, disease duration, family history of epilepsy, and disorganized VEEG background activity.

**Results:**

There were 17 (17.3%) refractory TLE‐MTS patients occurring PD after CAH, including 8 (8.2%) mood disorders, 7 (7.1%) anxiety disorders, 8 (8.2%) psychoses, and 1 (1.0%) interictal dysphoric disorder. Employed status correlated with low PD occurrence, while disease duration and asymmetric VEEG background activity positively correlated with PD occurrence. Multivariate logistic analysis revealed employed status (*P* = 0.009) could independently predict lower PD occurrence, while highest education (*P* = 0.027), disease duration (*P* = 0.028), seizure frequencies (*P* = 0.015), and asymmetric VEEG background activity (*P* = 0.034) could independently predict higher PD occurrence. Receiver operating characteristic curve showed combination of these five factors (area under curve (AUC) = 0.871, 95%CI: 0.783‐0.960) disclosed a great predictive value of PD occurrence. The sensitivity and specificity were 70.6% and 92.6% at the best cutoff point. In addition, the percentage of PD was increased with higher Engel classification (*P* = 0.003).

**Conclusion:**

Employed status, highest education, disease duration, seizure frequencies, and asymmetric VEEG background activity correlate with PD occurrence independently in epileptic patients.

## 1 INTRODUCTION

Epilepsy, a transient occurrence of signs and/or symptoms owning to abnormal excessive or synchronous neuronal activity in the brain, is a kind of chronic disorder that frequently causes long‐term medical, psychological, and social sequelae.^1^, ^2^ According to the report of World Health Organization (WHO), there are estimated more than 50 million epilepsy patients and 2.4 million new cases occurring worldwide per year.^3^ For adult epilepsy patients, approximately 60% patients are diagnosed as partial‐onset epilepsy, whose most common subtype is temporal lobe epilepsy (TLE), and mesial temporal sclerosis (MTS) is considered as the most common pathological substrate of TLE.^4^, ^5^ For these TLS‐MTS patients, most of them have been reported to be refractory to antiepileptic drugs (AEDs), but have favorable responses to surgery, including cortico‐amygdalohippocampectomy (CAH), which is a type of anterior and mesial temporal lobectomy (ATL) and one of optimal choices in patients with refractory epilepsy.^5^, ^6^

Although epilepsy surgery contributes to estimated 60%‐70% remission rate for long‐term clinical symptoms, the quality of life (QoL) is still difficult to be improved, partially due to high prevalence rate of psychiatric comorbidity in TLE‐MTS patients. There are about 20%‐40% TLE‐MTS patients combining with psychiatric disorders (PD), and more than 70% refractory patients also occur this seizure disorder.^7^, ^8^, ^9^, ^10^, ^11^, ^12^ High prevalence of PD might contribute to worse outcomes of surgery in refractory TLE‐MTS patients. Thus, more understanding about the presurgical factors predicting PD in refractory TLE‐MTS patients underwent CAH is necessary.

The risk factors related to PD in epilepsy patients after surgery have raised concern, and some researchers suggest that several sociodemographic and clinical factors are correlated with PD among epilepsy patients, including employed status, seizure‐free period, and marital problems.^13^, ^14^ However, little is known about the pre‐surgical predictors for PD in refractory TLE‐MTS patients received CAH. Thus, the purpose of this study was to evaluate the predictive value of presurgical factors for PD in refractory TLE‐MTS patients underwent CAH.

## 2 MATERIALS AND METHODS

### 2.1 Participants

A total of 98 refractory TLE‐MTS patients underwent CAH between January 2007 and December 2015 at Department of Neurosurgery in Hospital of Chengdu Office of People's Government of Tibetan Autonomous Region were consecutively enrolled in this cohort study. The inclusion criteria were as follows: (a) Diagnosed as refractory TLE according to International League Against Epilepsy (ILAE) classification.^15^ (b) Complicated with unilateral MTS by MRI findings. (c) Age at surgery above 18 years. (d) Without presurgical PD. (e) Followed‐up duration above 1 year. The exclusion criteria were as follows: (a) Complicated with clinical or other neurological illnesses besides epilepsy. (b) Complicated with cognitive impairments precluding psychiatric and clinical evaluations. (c) Complicated with bilateral MTS. (d) Complicated with other epilepsy syndromes and antihistamine administration or alcohol consumption within 72 hours prior to the psychiatric evaluation. (e) Pregnant or breast‐feeding female patients. This study was approved by the Ethics Committee of Hospital of Chengdu Office of People's Government of Tibetan Autonomous Region. All patients or their guardians provided the written informed consents.

### 2.2 Procedures

Before CAH, patients received continuous video‐electroencephalographic (VEEG) monitoring with 32‐channel EEG recording for two‐six days, accompanied with electrodes placed according to the 10‐10 system on the temporal lobe, including sphenoidal electrodes. The CAH procedure consisted of an en block resection of the superior, middle, inferior temporal, and fusiform gyri, with a posterior limit of 4.5 cm from the tip of the temporal lobe, and after opening the temporal horn, the mesial temporal structures (hippocampus, amygdala, and parahippocampal gyrus) were also resected as described in previous studies.^16^, ^17^

### 2.3 Data collection

Comprehensive factors preoperation were recorded including age at surgery, gender, married status, employment status, highest education, history of smoke, history of drink, age at epilepsy onset, disease duration, family history of epilepsy, family history of PD, seizure frequencies, presence of febrile seizures, presence of left‐sided MTS, disorganized VEEG background activity, asymmetric VEEG background activity, contralateral slow waves on VEEG, and contralateral epileptiform discharges on VEEG.

### 2.4 Assessments and definitions

(a) Refractoriness to medical treatment was defined as seizures persisted after the utilization of at least two first‐line medications for partial seizures at the highest tolerated doses for at least 6 months. (b) MTS was defined as atrophy, an increase in T2‐weighted signal, a decrease in T1‐weighted signal, and a disrupted internal structure of the hippocampus were present, accompanied by atrophy of the amygdala and/or temporal pole signal alteration by MRI imaging; mesial temporal lobe epilepsy (MTLE) is a chronic disorder of the nervous system characterized by recurrent, unprovoked focal seizures that arise in the hippocampus, the parahippocampal gyrus, and the amygdala which are located in the inner (medial) aspect of the temporal lobe.^18^ (c) According to the International Federation of Societies for Electroencephalography and Clinical Neurophysiology (IFSECN), epileptiform discharges are defined as "distinctive waves or complexes, distinguished from background activity, and resembling those recorded in a proportion of human subjects suffering from epileptic disorders….," while this somewhat circular definition makes clear that criteria must be verified empirically.^19^ (d) The treatment efficiency was assessed according to Engel classification; after CAH, the Engel classification scales are listed in (Table 1).^20^, ^21^ (e) PD were diagnosed according to Diagnostic and Statistical Manual of Mental Disorders (DSM‐IV) Axis I criteria in the follow‐up duration with the last follow‐up date March 2017.^22^ In addition, other epilepsy commonly related to PD such as psychoses and interictal dysphoric disorder, not covered by the DSM‐IV, was also assessed using the ILAE criteria.^23^

**Table 1:** Engel classification scales

Class I	No disabling seizures
A	Completely seizure‐since surgery
B	Nondisabling simple partial seizures only
C	Some disabling seizures after surgery, but free of disabling seizures for at least 2 y
D	Generalized convulsion with antiepileptic drug withdrawal only
Class II	Rare disabling seizures
A	Initially free of disabling seizures but has rare disabling seizures now
B	Rare disabling seizures since surgery
C	More than rare disabling seizures since surgery, but rare seizures for at least 2 y
D	Nocturnal seizures only
Class III	Worthwhile improvement
A	Worthwhile seizure reduction
B	Prolonged seizure‐free intervals but less than 2 y
Class IV	No worthwhile improvement
A	Significant seizure reduction
B	No appreciable change in seizure frequency
C	Seizures are frequent or worse

### 2.5 Statistics

Statistical analysis was performed using SPSS 22.0 software (IBM, Armonk, NY) and Graphpad Prism 6 software (GraphPad Software Inc, New York, NY, USA). Data were presented as mean ± SD or count (percentage). Comparison was determined by *t* test, chi‐square test, or Wilcoxon rank sum test. Baseline characteristics affecting PD occurrence were determined by univariate logistic regression analysis, while all factors with *P* value no more than 0.1 were further detected by multivariate logistic regression analysis. Furthermore, receiver operating characteristic (ROC) curve was used to assess the predictive value of the factors affecting PD occurrence. *P* value <0.05 was considered significant.

## 3 RESULTS

### 3.1 Baseline characteristics

Mean age of these 98 refractory TLE‐MTS patients underwent CAH was 35.95 ± 10.47 years, and the number of male and female was 48 and 50, respectively (Table 2). As to employment status, 76 (77.6%) patients were employed, while 22 (22.4%) patients were unemployed. The mean age at epilepsy onset was 10.63 ± 6.80 years, and the mean value of disease duration was 25.32 ± 8.41 years. The number of patients with family history of epilepsy and family history of psychiatric disorders was 25 (25.5%) and 14 (14.3%), respectively. There were 26 (26.5%), 21 (21.4%), 25 (25.5%), and 26 (26.6%) patients, respectively, with disorganized VEEG background activity, asymmetric VEEG background activity, contralateral slow waves on VEEG, and contralateral epileptiform discharges on VEEG. Other baseline characteristics are shown in Table 2.

**Table 2:** Baseline characteristics of refractory TLE‐MTS patients

Items	Refractory TLE‐MTS patients (N = 98)
Age at surgery (years)	35.95 ± 10.47
Gender (male/female)	48/50
Married status (n/%)	
Married	63 (64.3)
Not married	35 (35.7)
Employment status (n/%)	
Employed	76 (77.6)
Unemployed	22 (22.4)
Highest education (n/%)	
Primary school or less	21 (21.4)
High school	42 (42.9)
Undergraduate	28 (28.6)
Graduate or above	7 (7.1)
History of smoke (n/%)	17 (17.3)
History of drink (n/%)	5 (5.1)
Age at epilepsy onset (years)	10.63 ± 6.80
Disease duration (years)	25.32 ± 8.41
Family history of epilepsy (n/%)	25 (25.5)
Family history of psychiatric disorders (n/%)	14 (14.3)
Seizure frequencies (times per month)	6.7 ± 1.7
Presence of febrile seizures (n/%)	12 (12.2)
Presence of left‐sided MTS (n/%)	65 (66.3)
Disorganized VEEG background activity (n/%)	26 (26.5)
Asymmetric VEEG background activity (n/%)	21 (21.4)
Contralateral slow waves on VEEG (n/%)	25 (25.5)
Contralateral epileptiform discharges on VEEG (n/%)	26 (26.6)

Data were presented as mean ± SD or count (percentage).

MTS, mesial temporal sclerosis; TLE, temporal lobe epilepsy; VEEG, video‐electroencephalographic.

### 3.2 Engel classification of postoperative outcome

Engel classification was used for the assessment of the treatment efficiency. As presented in Table 3, the number of refractory TLE‐MTS patients with Engel classification Classes I, II, III, and IV was 73 (74.5%), 17 (17.3%), 6 (6.1%), and 2 (2.1%), respectively.

**Table 3:** Classification of postoperative outcome (Engel classification)

Engel classification	Patients (n/%)
Class I	73 (74.5)
Class II	17 (17.3)
Class III	6 (6.1)
Class IV	2 (2.1)

Data were presented as count (percentage).

### 3.3 Occurrence of PD after CAH

There were 17 (17.3%) refractory TLE‐MTS patients occurring PD after CAH (Table 4). Among these, the numbers of patients with mood disorder, anxiety disorder, psychoses, and interictal dysphoric disorder were 8 (8.2%), 7 (7.1%), 8 (8.2%), and 1 (1.0%), respectively (The subjects may suffer from more than one disorder).

**Table 4:** Occurrence of PD after surgery

Items	Patients (n/%)
PD	17 (17.3)
Mood disorders	8 (8.2)
Anxiety disorders	7 (7.1)
Psychoses	8 (8.2)
Interictal dysphoric disorder	1 (1.0)

The subjects may suffer from more than one disorder. Data were presented as count (percentage).

PD, psychiatric disorders.

### 3.4 Comparison of baseline characteristics between PD patients and non‐PD patients

After CAH, there were 17 (17.3%) PD patients and 81 (82.6%) non‐PD patients. PD patients had lower percentage of employment (58.8% vs 81.5%, *P* = 0.046), longer disease duration (30.18 ± 8.59 years vs 24.30 ± 8.05 years, *P* = 0.008), and higher percentage of asymmetric VEEG background activity (47.1% vs 16.0%, *P* = 0.005) compared to non‐PD patients (Table 5). However, no difference was found in other baseline characteristics between PD patients and non‐PD patients (All *P* > 0.05).

**Table 5:** Comparison of baseline characteristics between PD patients and non‐PD patients

Items	PD patients (N = 17)	Non‐PD patients (N = 81)	*P* value
Age at surgery (years)	39.53 ± 9.14	35.20 ± 10.63	0.122
Gender (male/female)	6/11	42/39	0.214
Married status (n/%)			
Married	9 (52.9)	54 (66.7)	0.283
Not married	8 (47.1)	27 (33.3)	
Employment status (n/%)			
Employed	10 (58.8)	66 (81.5)	**0.046**
Unemployed	7 (41.2)	15 (18.5)	
Highest education (n/%)			
Primary school or less	1 (5.9)	20 (24.7)	0.129
High school	6 (35.2)	36 (44.4)	
Undergraduate	8 (47.1)	20 (24.7)	
Graduate or above	2 (11.8)	5 (6.2)	
History of smoke (n/%)	3 (17.6)	14 (17.3)	0.971
History of drink (n/%)	1 (5.9)	4 (4.9)	0.872
Age at epilepsy onset (years)	9.35 ± 5.45	10.90 ± 7.95	0.396
Disease duration (years)	30.18 ± 8.59	24.30 ± 8.05	**0.008**
Family history of epilepsy (n/%)	7 (41.2)	18 (22.2)	0.103
Family history of psychiatric disorders (n/%)	3 (17.6)	11 (13.6)	0.664
Seizure frequencies (times per month)	7.8 ± 2.5	6.4 ± 1.4	0.052
Presence of febrile seizures (n/%)	3 (17.6)	9 (11.1)	0.733
Presence of left‐sided MTS (n/%)	12 (70.6)	53 (65.4)	0.683
Disorganized VEEG background activity (n/%)	6 (35.3)	20 (24.7)	0.368
Asymmetric VEEG background activity (n/%)	8 (47.1)	13 (16.0)	**0.005**
Contralateral slow waves on VEEG (n/%)	3 (17.6)	22 (27.2)	0.413
Contralateral epileptiform discharges on VEEG (n/%)	4 (23.5)	22 (27.2)	0.758

Data were presented as mean ± SD or count (percentage). Comparison was determined by t test or chi‐square test.

PD, psychiatric disorders; MTS, mesial temporal sclerosis; VEEG, video‐electroencephalographic.

### 3.5 Baseline characteristics affecting PD occurrence after CAH by logistic regression model analysis

Univariate logistic regression analysis was performed to evaluate baseline characteristics affecting PD occurrence in refractory TLE‐MTS patients underwent CAH (Table 6). Employed status (*P* = 0.048) was associated with lower PD occurrence, while highest education (*P* = 0.027), disease duration (*P* = 0.012), seizure frequencies (*P* = 0.014), and asymmetric VEEG background activity (*P* = 0.007) were correlated with higher PD occurrence in refractory TLE‐MTS patients underwent CAH. All factors with *P* value no more than 0.1 were further detected by multivariate logistic regression analysis, which revealed that employed status (*P* = 0.009) could independently predict lower PD occurrence, while highest education (*P* = 0.027), disease duration (*P* = 0.028), seizure frequencies (*P* = 0.015), and asymmetric VEEG background activity (*P* = 0.034) were independent factors for predicting higher PD occurrence in refractory TLE‐MTS patients underwent CAH.

**Table 6:** Baseline characteristics affecting PD occurrence by logistic regression model analysis

Parameters	Univariate logistic regression				Multivariate logistic regression			
	*P* value	OR	95% CI		*P* value	OR	95% CI	
			Lower	Higher			Lower	Higher
Age at surgery	0.125	1.041	0.989	1.096	‐	‐	‐	‐
Gender (male)	0.220	0.506	0.171	1.500	‐	‐	‐	‐
Married status (married)	0.287	0.563	0.195	1.621	‐	‐	‐	‐
Employment status (employed)	**0.048**	0.325	0.106	0.992	**0.009**	0.133	0.029	0.610
Highest education	**0.027**	2.050	1.084	3.875	**0.027**	2.355	1.103	5.027
History of smoke	0.971	1.026	0.260	4.050	‐	‐	‐	‐
History of drink	0.872	1.203	0.126	11.489	‐	‐	‐	‐
Age at epilepsy onset	0.394	0.963	0.884	1.050	‐	‐	‐	‐
Disease duration	**0.012**	1.093	1.020	1.171	**0.028**	1.098	1.010	1.194
Family history of epilepsy	0.110	2.450	0.817	7.351	‐	‐	‐	‐
Family history of psychiatric disorders	0.664	1.364	0.336	5.528	‐	‐	‐	‐
Seizure frequencies	**0.014**	1.431	1.075	1.903	**0.015**	1.507	1.083	2.097
Presence of febrile seizures	0.459	1.714	0.412	7.139	‐	‐	‐	‐
Presence of left‐sided MTS	0.683	1.268	0.406	3.962	‐	‐	‐	‐
Disorganized VEEG background activity	0.371	1.664	0.545	5.076	‐	‐	‐	‐
Asymmetric VEEG background activity	**0.007**	4.650	1.514	14.280	**0.034**	4.951	1.128	21.735
Contralateral slow waves on VEEG	0.418	0.575	0.151	2.194	‐	‐	‐	‐
Contralateral epileptiform discharges on VEEG	0.758	0.825	0.243	2.803	‐	‐	‐	‐

Data were presented as *P* value, OR (odds ratio), and 95% CI (confidence interval). Baseline characteristics affecting PD occurrence were determined by univariate logistic regression analysis, while all factors with *P* value no more than 0.1 were further detected by multivariate logistic regression analysis.

Highest education was scored as: primary school or less = 1, high school = 2, undergraduate = 3, graduate, or above = 4. *P* Value <0.05 was considered significant.

MTS, mesial temporal sclerosis; PD, psychiatric disorders; VEEG, video‐electroencephalographic.

### 3.6 Predictive value of factors for risk of PD after CAH

**Figure 1:**
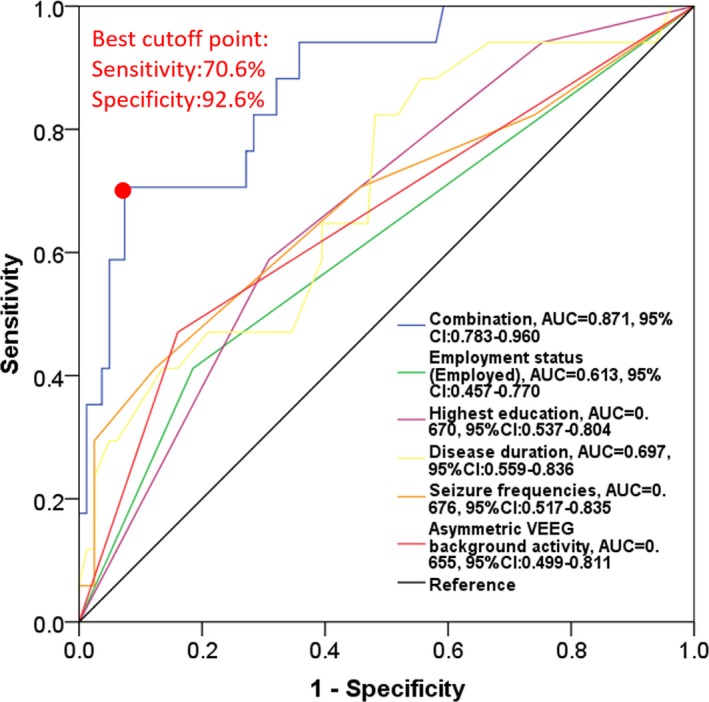
Predictive value of factors in the risk of PD after CAH. The combination of employed status, highest education, disease duration, seizure frequencies, and asymmetric VEEG background activity disclosed a great predictive value of PD occurrence in refractory TLE‐MTS patients underwent CAH. The sensitivity and specificity were 70.6% and 92.6% at the best cutoff point. Receiver operating characteristic (ROC) curve was used to assess the predictive value of the factors affecting PD occurrence. CAH, cortico‐amygdalohippocampectomy; PD, psychiatric disorders; TLE‐MTS, temporal lobe epilepsy and mesial temporal sclerosis

According to the results of multivariate logistic regression analysis, there were five independent factors predicting PD occurrence in refractory TLE‐MTS patients underwent CAH, including employed status, highest education, disease duration, seizure frequencies, and asymmetric VEEG background activity. We further performed ROC curves to assess the predictive value of combination of these five factors for PD occurrence in refractory TLE‐MTS patients underwent CAH. As presented in Figure 1, the area under curve (AUC) in ROC curves of employed status, highest education, disease duration, seizure frequencies and asymmetric VEEG background activity was as follows: 0.613, 95%CI: 0.457‐0.770), 0.670 (95%CI: 0.537‐0.804), 0.697 (95%CI: 0.559‐0.836), 0.676 (95%CI: 0.517‐0.811), and 0.655 (95%CI: 0.499‐0.811), respectively. In addition, the combination of these five factors disclosed a great predictive value for PD occurrence in refractory TLE‐MTS patients underwent CAH (AUC = 0.871, 95%CI: 0.783‐0.960). The sensitivity and specificity were 70.6% and 92.6% at the best cutoff point, which was defined as the point with the max value of sensitivity plus specificity.

### 3.7 Correlation of PD occurrence after CAH with Engel classification

**Figure 2:**
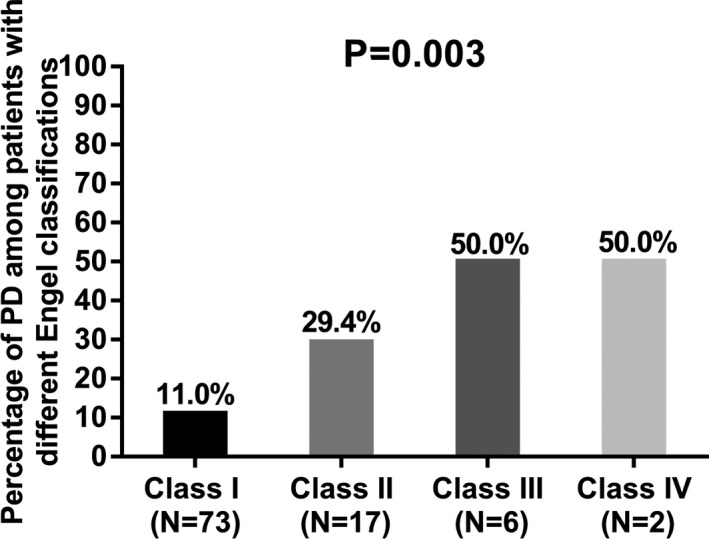
Correlation of PD occurrence after CAH with Engel classification. The occurrence of PD was correlated with increased Engel classification Comparison was determined by Wilcoxon rank sum test. *P* value < 0.05 was considered significant. CAH, cortico‐amygdalohippocampectomy; PD, psychiatric disorders; TLE‐MTS, temporal lobe epilepsy and mesial temporal sclerosis

In order to further evaluate the influence of treatment efficacy on occurrence of PD in refractory TLE‐MTS patients underwent CAH, we performed Wilcoxon rank sum test, which revealed that the occurrence of PD was correlated with increased Engel classification (*P* = 0.003), and the occurrence of PD among refractory TLE‐MTS patients with Engel classification Class I, Class II, Class II, and Class IV were 11.0%, 29.4%, 50.0%, and 50.0%, respectively (Figure 2).

## 4 DISCUSSION

In the present study, we observed that: Firstly, there were 17 (17.3%) refractory TLE‐MTS patients occurring PD after CAH, including 8 (8.2%) mood disorders, 7 (7.1%) anxiety disorders, 8 (8.2%) psychoses, and 1 (1.0%) interictal dysphoric disorder. Secondly, employed status independently correlated with lower PD occurrence, while highest education, disease duration, seizure frequencies, and asymmetric VEEG background activity were independently associated with higher PD occurrence in refractory TLE‐MTS patients underwent CAH.

Refractory TLE‐MTS patients have been reported to have a high risk of psychiatric complications either before or after treatment. Previous studies have reported that there are approximately 20%‐40% TLE patients and estimated 70% refractory epilepsy patients suffering from PD. Among these, depression (24%‐74%), anxiety (10%‐25%), psychoses (2%‐9%), and personality disorders (1%‐2%) are commonly diagnosed PD.^7^, ^8^, ^13^, ^24^ Most of these studies focus on a total PD occurrence in epilepsy patients, while few studies have investigated PD occurrence after CAH in refractory TLE‐MTS patients, excluding presurgery PD occurrence. Hence, in order to decrease confounding effects from presurgical PD and more exactly assess PD occurrence after CAH, this cohort study enrolled 98 refractory TLE‐MTS patients underwent CAH, and all of them were without presurgical PD. We found that there were 17 (17.3%) refractory TLE‐MTS patients occurring PD after CAH, including 8 (8.2%) mood disorders, 7 (7.1%) anxiety disorders, 8 (8.2%) psychoses, and 1 (1.0%) interictal dysphoric disorder, which was relatively lower compared to previous studies. The possible reason was that the PD occurrence in this study was postsurgery PD, which has eliminated presurgical PD occurrence in refractory TLE‐MTS patients underwent CAH.

To date, many studies have investigated the predictive value of sociodemographic and clinical factors for PD occurrence in ellipse patients. An interesting study discloses that uncontrolled seizures and polytherapy, sense of stigma, and less family support are predictors of high occurrence of anxiety and depressive disorders in epilepsy patients.^25^ Meanwhile, short‐term seizure‐free period and employed status also have been reported to predict the presence of psychiatric disorders, including depression, anxiety, and psychoses in epilepsy patients.^14^ Another retrospective study discloses an obvious association of a reduced cognitive function with a pejorative psychiatric outcome after the operation in adult patients with MTLE associated with hippocampal sclerosis (HS) that is the most common focal, drug‐resistant epilepsy syndrome.^26^ Furthermore, other studies indicate that the stresses of family members correlate with the increased risk of behavior problems and psychological disorders in epilepsy patients.^27^, ^28^ Therefore, these previous results identify that several sociodemographic and clinical factors serve as predictive factors for PD in epilepsy patients. In the present study, we observed that employed status could independently predict lower PD occurrence, but highest education, disease duration, seizure frequencies, and asymmetric VEEG background activity were independently factors predicting higher PD occurrence in refractory TLE‐MTS patients underwent CAH. The possible reasons were as follows: (a) For employed status, considered as a social advantage, it could provide a steady income to decrease finance pressure for treatment of a chronic disease such as epilepsy, thereby reducing psychological burden to decrease the risk of PD occurrence to some extent. (b) For highest education, refractory TLE‐MTS patients underwent CAH who have higher education might be difficult to accept their disease conditions and sequelae, generating huge mental stress, thereby decreasing their confidence and increasing the risk of PD. (c) For disease duration, seizure frequencies, and asymmetric VEEG background activity, worse disease severity not only increases patients’ physiological pain, but also is considered as a worrisome shade for them to cause feeling of helplessness because the illness still occurs in spite of medications, thereby increasing the risk of PD occurrence, such as depression or anxiety. Therefore, all of employed status, highest education, disease duration, seizure frequencies, and asymmetric VEEG background activity factors could affect PD occurrence in refractory TLE‐MTS patients underwent CAH.

In order to know the effects of the combination of these five factors on PD occurrence in refractory TLE‐MTS patients underwent CAH, we further performed ROC curves and we observed that combination of employed status, highest education, disease duration, seizure frequencies, and asymmetric VEEG background activity factors disclosed a great predictive value of PD occurrence in refractory TLE‐MTS patients underwent CAH. The possible reasons were as follow: Each one of employed status, highest education, disease duration, seizure frequencies, and asymmetric VEEG background activity factors would affect patients’ minds to face a chronic disease, such as refractory TLE‐MTS, affecting their attitude for the treatment and complications to decrease/increase the PD occurrence, thus, the combination of these five factors might be considered as more accurate factor predicting PD occurrence in refractory TLE‐MTS patients underwent CAH. These results in our study might increase the social awareness about epilepsy and decrease the occurrence of PD in refractory TLE‐MTS patients underwent CAH.

Although some interesting results were found in this study, some limitations still existed. Firstly, sample size in this cohort study was relatively small, which only enrolled 98 refractory TLE‐MTS patients underwent CAH; further study with a large sample size was necessary. Secondly, all patients recruited in our study were from monocentric, thus, more patients from multicentre are needed to be enrolled in additional study. Thirdly, these patients had only MTS findings on their MRI, and these patients might have dual pathology.

In conclusion, employed status, highest education, disease duration, seizure frequencies, and asymmetric VEEG background activity correlate with PD occurrence independently in epileptic patients.
